# Retrograde and anterograde trans-synaptic viral tracing of neuronal connections reveals local and distant effects of ischemic stroke on dendritic spines

**DOI:** 10.1177/0271678X251345360

**Published:** 2025-05-25

**Authors:** Myrthe Van Sprengel, Jenna Butterworth, Patrick L Reeson, Craig E Brown

**Affiliations:** 1 Division of Medical Sciences, University of Victoria, Victoria, BC, Canada; 2 Department of Psychiatry, University of British Columbia, Vancouver, BC, Canada

**Keywords:** Diaschisis, connectome, dendritic spines, functional recovery, plasticity

## Abstract

Focal stroke leads to complex neurological disturbances with variable recovery. One explanation for this variability is that stroke disrupts local and remote neural circuits via the connectome, termed ‘diaschisis’. Past studies have yielded mixed effects of stroke on dendritic structure in distant regions. However, a previous limitation was the lack of sampling specifically from neurons directly connected to those within the infarct. To overcome this, we used retrograde and anterograde trans-synaptic AAVs to examine dendritic spine density in neurons that provide inputs to, or receive outputs (pre- and post-synaptic) from primary forelimb somatosensory cortex at 1 or 6 weeks after stroke. For both pre- and post-synaptic neurons, spine density was generally lower in superficial and deep neurons in peri-infarct and motor cortex at 1 week, which recovered by 6 weeks. By contrast, no changes in spine density were observed in ipsilateral secondary somatosensory (S2) or contralateral primary somatosensory cortex at 1 week, although there was an increase in spines in select S2 neurons at 6 weeks. Our data show that some cortical connections are more disrupted by stroke than others, particularly those in peri-infarct and motor cortex which could serve as an important substrate for stroke recovery and future therapies.

## Introduction

Stroke is a cerebrovascular disease that results in a rapid loss of function as a result of cell death in the ischemic core. However, functional impairments are usually complex and distributed; and are not fully explained by the location of the infarct or its size.^1,2^ Furthermore, the prognosis for recovery is quite variable and it is estimated that greater than 75% of survivors will live with chronic disability. One explanation is that distant, but connected brain regions are variably impacted by stroke damage.^3,4^ This idea known as *diaschisis* was postulated over a hundred years ago^5^ and has intuitive appeal since we now recognize that virtually any behavior requires communication between functionally defined cortical modules. For example, the “simple” motor task of reaching for and grasping a cup of coffee requires constant input and dynamic feedback from somatosensory systems that provide tactile and proprioceptive information to guide movement. It is therefore not surprising that when stroke patients suffer damage to the somatosensory cortex, there are significant motor impairments that variably recover over time.

Our understanding of diaschisis has improved and evolved over the years. For example, functional studies using electrophysiology, fMRI, radio-labelled glucose or optical imaging have shown that ischemic damage in one hemisphere usually dampens neural and metabolic activity in sensory-motor, limbic cortices of the ipsilesional hemisphere while augmenting activity in the contralateral hemisphere during the acute stage of recovery.^3,6–12^ Of note, brain activity patterns can partially return to normal over several weeks to months which correlates with the recovery of neurological function.^13–15^ The structures that underlie these functional changes during stroke recovery are varied and still being elucidated.^16^ Since the majority of synapses in the cortex terminate onto dendritic spines,^17–19^ a common approach to understanding the effects of stroke on individual neurons is to examine dendritic spine density. Indeed, there are many previous reports that have examined dendritic spine density in peri-infarct regions and distant sites. These studies generally agree that peri-infarct neurons show a loss of spines in the first few days to week after stroke,^20–24^ but in more distant regions like the contralateral hemisphere, the results are quite mixed. For example in the contralateral cortex, some show no effect of focal stroke or injury on spine density or turnover,^25,26^ whereas others indicate an increase or decrease in spine density.^27–30^

One caveat with previous anatomical studies is that they randomly sampled neurons (using Golgi-Cox, intracellular dye filling etc.) in functionally relevant regions (e.g. homotopic contralateral somatosensory cortex, ipsilateral motor cortex) without necessarily knowing if these neurons were in fact, directly connected to the stroke site. This is a credible concern given that each brain region sends and receives inputs from multiple regions,^31,32^ many of which would not be directly damaged by stroke. Thus, an important but unresolved question is whether cortical neurons with direct connections to the stroke site are more vulnerable/disrupted (or conversely resilient) to the effects of focal stroke? Since there are now tools available for modulating neural circuits *in vivo*, resolving this question could help inform or guide future neuro-modulatory therapies that target specific circuits for improving stroke recovery. Over the past decade, viral tracing tools have been developed that allow retrograde and trans-synaptic labelling of neurons pre- or post-synaptic to cortical neurons located within a specific region.^33,34^ Using this technology, our goal was to assess dendritic spine density in superficial and deep neurons in local and remotely connected brain regions after ischemic cortical stroke. Our results suggest that not all connected cortical regions are equally affected by stroke, with neurons in peri-infarct and motor cortex being particularly sensitive and/or plastic.

## Materials and methods

### Animals

Two to four month old male and female adult mice were used in this study. Mice were randomly assigned to one of the three experimental groups. For retrograde AAV-GFP based tracing, all mice used were C57BL/6J. Based on sample sizes and expected variances from our previous studies examining dendritic spine plasticity after stroke,^22,35^ we collected data from 11 sham mice (5 male, 6 female), 8 mice at one week stroke (4 male, 4 female) and 5 mice at 6 weeks recovery (2 male, 3 female). Two additional male C57BL/6J mice were used for assessing survival of retrogradely labelled neurons after stroke. Mice expressing cre-dependent tdtomato and GCAmP6s (Ai9:Ai162D, JAX strains #007909 and #031562, respectively) were used for AAV based trans-synaptic labelling of neurons that receive outputs from forelimb primary somatosensory (FLS1) cortex. For these experiments, we collected data from 9 sham mice (4 male, 5 female), 10 mice at one week stroke (5 male, 5 female) and 5 mice at six weeks post-stroke (3 male, 2 female). Although we attempted to balance the number of males and females within each group, our study was not sufficiently powered to detect sex differences and thus all data are presented with sexes pooled. The mice were housed in standard cages with unlimited access to water and a standard lab diet. The room was humidity controlled and maintained at 22.5 °C ± 2.5 °C, with a 12-hour light/dark cycle. All animals used for these experiments followed protocols approved by the University of Victoria Animal Care Committee and guidelines set by the Canadian Council on Animal Care (CCAC). Reporting of this work complies with ARRIVE guidelines.

### Viral tracing of pre- or post-synaptic neurons to/from FLS1 cortex

Mice were anesthetized with isoflurane mixed in medial air (2% induction, 1.3% maintenance, 0.7 L/min flow) until sufficiently sedated and then fitted into a surgical stage. Animals were kept on a heating pad and body temperature was maintained at 37 °C using a rectal thermoprobe and temperature feedback regulator. A subcutaneous injection of lidocaine under the scalp was administered to reduce pain during/after the surgery. A midline incision was made along the scalp, and clamps were used to hold the skin back exposing the skull. A high-speed dental drill created a hole centered above the right forelimb primary somatosensory (FLS1) cortex, using the following coordinates: 0 mm anterior and 2.5 mm lateral from bregma.^36^ Using a stereotaxic arm, a 32-gauge Hamilton syringe or glass micropipette (tip diameter 25–40 µm) was then used to slowly inject 0.1–0.4 μL of either retro pAAV.CAG.GFP (Addgene 37825-AAVrg, Titre: 2.2 × 10^13^ GC/mL; serotype: AAVrg) or trans-synaptic pENN.AAV.hSyn.Cre.WPRE.hGH (Addgene 105553, Titre: 2.6 × 10^13^ GC/mL, serotype: AAV1) diluted at 1:10 or 1:2 in sterile 0.1 M PBS, respectively. Following the injection, mice recovered from the anesthetic and were monitored on a warming pad before being returned to their home cage.

### Photothrombotic stroke

Three weeks after the intracortical injection of AAV, a photothrombotic stroke was targeted to the same site. Briefly, mice were anesthetized with isoflurane mixed in medical air (2% induction, 1.3% maintenance, 0.7 L/min flow) and then fitted into a surgical stage. Body temperature was maintained at 37 °C and lidocaine was injected under the scalp for topical analgesia. Following a midline incision, clamps were used to hold the skin back to expose the skull. A high-speed dental drill was used to thin the skull above FLS1 cortex where we previously injected AAV (thinned a 2.25 mm^2^ area until surface vessels became visible). Rose Bengal dye was dissolved in sterile filtered 0.1 M PBS and injected at a dose of 100 mg/kg. The thinned skull was then illuminated by a green laser (532 nm wavelength, ∼8–10 mW) for 5–15 minutes until the surface vessels stopped flowing. Sham control mice received either the injection of rose bengal without the green light, or green light without rose bengal. After the procedure, animals recovered on a warming pad then were returned to their home cage.

### Tissue processing

At 1 or 6 weeks after sham surgery or stroke procedure, mice were overdosed with isoflurane or sodium pentobarbital and transcardially perfused with 6–10 mL of 0.1 M PBS followed by 4% paraformaldehyde (PFA). The brains were extracted and preserved in 4% PFA overnight, and then transferred to 0.1 M PBS + 0.02% sodium azide solution. The brains were then mounted and sliced on a vibratome (Leica VT1200S) in 100 μm thick coronal sections and stored in a 12-well plate with 0.1 M PBS + 0.02% azide. The sections were mounted onto gelatin-coated slides, dried and coverslipped with Fluromount-G (Southern Biotech).

### Imaging

To determine the volume of ischemic damage as well as the position of neurons relative to the cortical surface (for determining superficial vs. deep) or the infarct border, low magnification images were collected with epifluorescence Olympus BX51 microscope equipped with a 4x objective lens (NA = 0.13) and a DP73 digital CCD camera coupled to CellSens software. The infarct region was easily identified with transmitted light and assessed from every 3^rd^ section (spaced 300 µm apart). The infarct area in each section was manually traced in Image J FIJI (ver. 1.53) and multiplied by the distance between sections to estimate infarct volume.

High-resolution imaging of dendritic spines was performed using either a confocal or 2-photon microscope. For retrogradely labelled GFP expressing neurons, image stacks were collected with an Olympus confocal microscope with a 60x oil objective lens (NA= 1.35). Confocal image stacks were collected with a 488 nm laser and GFP filter set using the following acquisition specifications: 211.2 × 211.2 µm (0.13 µm/pixel) region with Kalman frame average of 2, z-step of 0.6 µm, and a pixel dwell time of 4.0 us/pixel. For trans-synaptically labelled neurons expressing tdTomato/GCaMP6s, a two-photon microscope was required to collect high resolution image stacks because confocal imaging created too much out of plane photo-bleaching of tdTomato signal. Thus a two-photon microscope (Olympus FV1000MPE) equipped with 60X water-immersion lens (LUMFL NA = 1.1) was used in conjunction with Olympus’ Fluoview FV10-ASW software to capture image stacks. Tissue specimens were excited with the MaiTai DeepSee Ti:sapphire femtosecond laser tuned to 945 nm and emitted light was collected with GFP and Texas Red filter sets. Acquisition parameters were as follows: 2 µs pixel dwell time; with 0.8 µm z-steps with Kalman filter of 2 covering an area of 211.2 × 211.2 µm (pixel size = 0.13 µm/pixel).

In each brain, we attempted to find a minimum of 5 neurons from each of four cortical regions to image. Brain regions were defined based on overlaying stereotaxic images from Paxinos and Franklin.^36^ Although we used stereotaxic coordinates to center AAV injections and stroke in the right FLS1 cortex, resultant labelling of neurons next to the injection site or stroke, would have involved neurons located in the FLS1, as well as a potion of primary hindlimb somatosensory (HLS1) cortex. Since neurons could not be imaged in the center of the AAV injection (in shams) due to extreme density of labelling and brightness, our analysis of neurons in peri-infarct or comparable sham region included both FLS1 and those in lateral edges of the HLS1. Thus, we binned measurements from neurons in both FLS1 and HLS1, which we hereafter refer to as “S1 cortex”. Given that imaging of subcortical and thalamic neurons was not possible due to weak or absent signal, or dense axonal overlap, only the ipsilateral motor (M1 and M2 combined), primary FL/HL somatosensory (S1), secondary somatosensory (S2), and contralateral S1 cortex were examined. Furthermore, although we could detect some neurons in contralateral motor or S2 cortex, labelling was too sparse and unreliable to image across mice and therefore not included in the analysis. Peri-infarct neurons were defined as those <800 µm from the medial or lateral edge of the infarct border, whereas motor cortex neurons had to be >800 µm from the medial infarct border.

To assess the survival of neurons connected to the infarct, two C57BL6 mice had a cranial imaging window installed as previously described^14^ and were micro-injected with retro pAAV.CAG.GFP. Three weeks later, mice were subjected to longitudinal two-photon imaging. Mice were lightly anesthetized with isoflurane (2% for induction and 1% for maintenance in medical air) and then fitted into a custom made apparatus for stabilizing the head. High-resolution two-photon image stacks of GFP labelled cortical neurons were acquired *in vivo* using an Olympus FV1000MPE laser scanning microscope fed by a mode-locked Ti:Sapphire laser source (Mai Tai XF DeepSee, Spectra-Physics) and equipped with a water-dipping 40× objective lens [Olympus; numerical aperture (NA) = 0.8]. The laser was tuned to 900 nm for excitation of GFP and emitted light was collected through an emission filter (495 to 540 nm). Images were acquired at 2 μm z-step intervals from a depth of 80–280 µm below the cortical surface, with each image covering an area of 325 × 325 μm (1024 × 1024 pixel sampling), averaging 2 frames per image. A total of 208 neurons were imaged in 4 areas from 2 mice, before photothrombotic stroke was targeted to the site of AAV injection, and then again 2 and 4 days afterwards.

### Data analysis

Similar to our previous studies^22,35^ quantification of dendritic spine density in sham or stroke affected animals was conducted in image stacks that were coded by another researcher to ensure blinding of experimental conditions. The blinded observer examined 3-dimensional image stacks of individual neurons and selected a segment of primary apical, secondary apical, primary basilar and secondary basilar dendrite in each neuron. The dendrites were selected by the following criteria: i) had bright fluorescent signal relative to background, ii) did not significantly overlap other dendrites or axons and iii) were at least 30 μm in consecutive length for analysis. To control for cell-to-cell differences in fluorescence intensity, the grey scale in image stacks was set to 40% of the maximal pixel intensity of the measured dendritic shaft (based on 4 pixel thick plot profile line). Spines were manually counted by two blinded observers (authors J.B. and M.V.S.) and included in the analysis if they protruded from the shaft a minimum of 0.5 µm (4 pixels) and extended no more than 4 μm (to exclude possibility of counting short branches). Since there are very few spines on dendrites right next to the soma, spine counting started 20 μm from the soma. For each neuron, approximately 300–400 µm of dendrite was analyzed. Manual spine counts for both pre-synaptic and post-synaptic neurons were validated by comparing blinded spine density estimates between the two observers. As shown in Supplementary Figure 1, spine density counts did not differ significantly between observers. Superficial neurons were defined as those with a soma <450 µm from pial surface, whereas deep cortical neurons were considered >450 µm (but not in white matter). This threshold was based on the anatomy of cortical layers where 450 µm would reliably distinguish neurons in layer 2/3 from those in deeper layers 5 and 6. It was also based on our analysis of neuronal depth from the cortical surface. Plotting the relative frequency of neurons shows two distributions with 450 µm representing the approximate point where the distributions meet (Supplementary Figure 2). In summary, the following parameters were recorded for each neuron: superficial or deep, type of dendrite (apical or basilar), branch order (primary or secondary), and the number of spines per segment of dendritic length.

### Exclusion criteria and statistics

Any mice that did not exhibit sufficient fluorescent labelling of cells (minimum 5 neurons in 3 out of 4 brain regions) were excluded from analysis. A summary of experimental mice and sources of attrition in this study is provided in Supplementary Table 1. All data were analysed using GraphPad Prism 10 software. Student’s *t* tests (unpaired) were used to analyze simple between group differences (e.g. infarct volume). Two-way ANOVAs were used to compare differences in spine density with main factors being: stroke (sham vs 1 vs 6 weeks), cortical region, depth (superficial vs deep neurons), or primary vs secondary dendrites. Significant main effects from ANOVAs were further analyzed with Tukey’s multiple comparisons tests. *P* values <0.05 were considered statistically significant.

## Results

### Viral based labelling of neurons pre and post-synaptic to FLS1

**Figure 1.**
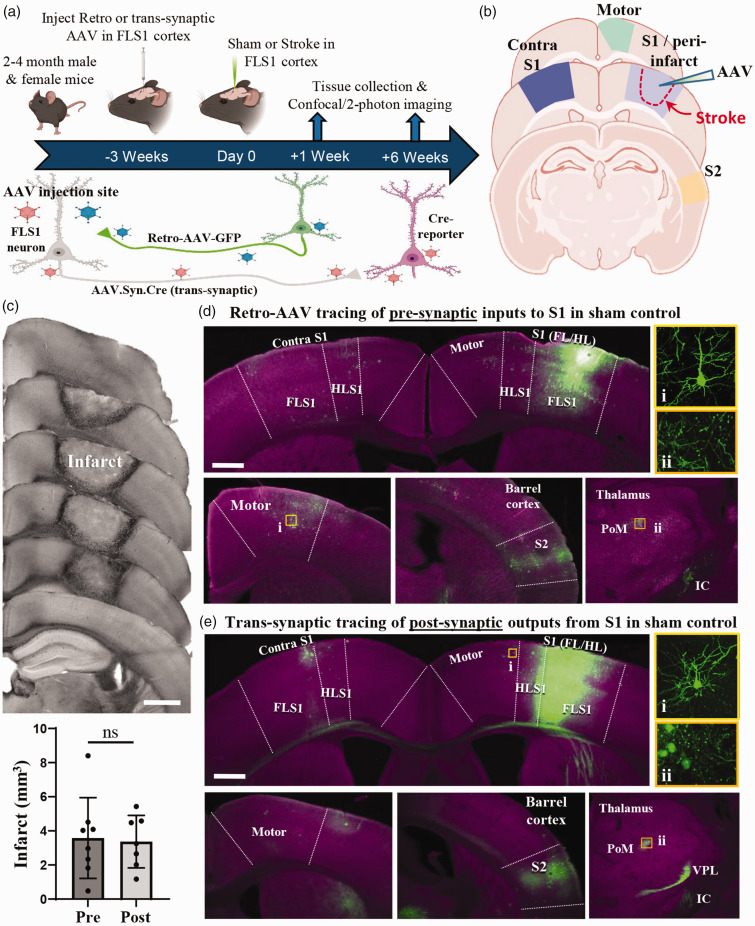
Retrograde or trans-synaptic tracing of pre and post-synaptic cortical connections. (a) Summary and timeline of experiments. (b) Schematic of coronal brain sections illustrating where neurons were sampled from. (c) Top: Transmitted light images of a representative photothrombotic stroke (at 1 week recovery), showing the anterior to posterior profile in coronal sections. Bottom: Graph shows individual and average infarct volume at 1 week post-stroke in both experimental groups. (d,e) Wide-field images reveal the distribution of fluorescently labeled pre-synaptic (d) or post-synaptic (e) neurons (green). Note that the majority of labelled neurons are located in ipsi and contra primary somatosensory (S1) cortex, ipsi Motor and secondary somatosensory (S2) cortex. Insets show higher magnification examples of labelled neurons in motor cortex or thalamus. FLS1: primary forelimb somatosensory cortex, HLS1: primary hindlimb somatosensory cortex, VPL: ventral-posterior lateral thalamus, PoM: posterior medial thalamus, IC: internal capsule. Data in (c) analysed by two-tailed unpaired t-test. Scale bars in c = 1 mm, d,e = 0.5 mm. ns: not-significant. Data presented as mean ± SD.

**Figure 2.**
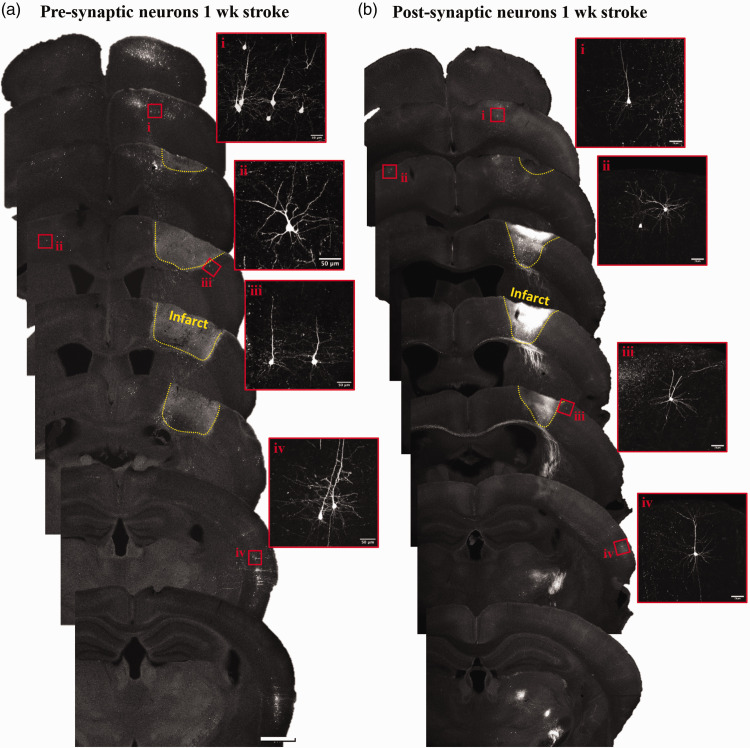
Distribution of labelled neurons 1 week after stroke. (a) Widefield fluorescent images show distribution of retrogradely labelled neurons in coronal sections arranged in an anterior to posterior manner. (b) Montage shows anterior to posterior distribution of trans-synaptically labelled neurons that remain 1 week after stroke. Note that bright signal within the infarct core was associated with auto-fluorescent debris (particularly bright in red channel) rather than cellular labelling. Insets show higher magnification images of neurons in select regions. Scale bar a,b = 1 mm, insets =50µm.

In order to understand stroke related diaschisis at the level of individual cortical connections, we micro-injected either retrograde or trans-synaptic anterograde AAVs into the right FLS1 cortex in adult male and female mice (Figure 1(a) and (b)). Mice then recovered for 1 or 6 weeks after sham procedure or photothrombotic stroke. We chose these early and late time points based on previous studies which have shown that limb function is maximally impaired in the first week after focal cortical stroke, but undergoes considerable improvement by 6 weeks recovery.^37–39^ As shown in Figure 1(c), ischemic damage extended from the cortical surface down to the white matter and occupied comparable volumes of 3.54 ± 1.15 mm^3^ or 3.36 ± 0.55 mm^3^ at 1 week recovery for experiments examining retrograde/pre-synaptic neurons or post-synaptic neurons, respectively. Assuming a hemispheric volume of 254.5 mm^3^ for an adult C57BL/6 mouse,^40^ the infarct volume comprised approximately 1.3% of the right hemisphere. Injection of retrograde or anterograde trans-synaptic AAVs allowed us to examine dendritic spine density in neurons that were either pre-synaptic or post-synaptic, respectively, to neurons in FLS1 cortex that would ultimately be destroyed by ischemic stroke. The regional distribution of fluorescently labelled neurons for sham controls (Figure 1(d) and (e)) and stroke affected mice (Figure 2(a) and (b)) was generally similar for both AAV tracing methods and consistent with previous tract tracing studies in FLS1 cortex.^6,41^ In particular, labelled neurons were identified in superficial and deep layers of the ipsilateral S1 (FLS1 and HLS1 treated as one), motor (M1 and M2 were treated as one) and secondary somatosensory (S2) cortex, as well as the contralateral S1 cortex (Figures 1(d) and (e) and 2(a) and (b)). As has been reported in previous work using these AAVs,^33^ not every connected brain region is well labelled due to viral tropisms. For example, fluorescent labelling of somatosensory thalamic nuclei (VPL and PoM) was either absent, weak (i.e. could not reliably track dendritic branches) or was obscured by dense axonal labelling (Figures 1(d) and (e) and 2(a) and (b)). Furthermore, although we could detect some neurons in contralateral motor or S2 cortex, labelling was too sparse and/or unreliable to image across mice. Due to these technical issues, our analysis was focused exclusively on cortical neurons in the following regions: ipsi and contra S1, motor and S2 cortex.

**Figure 3.**
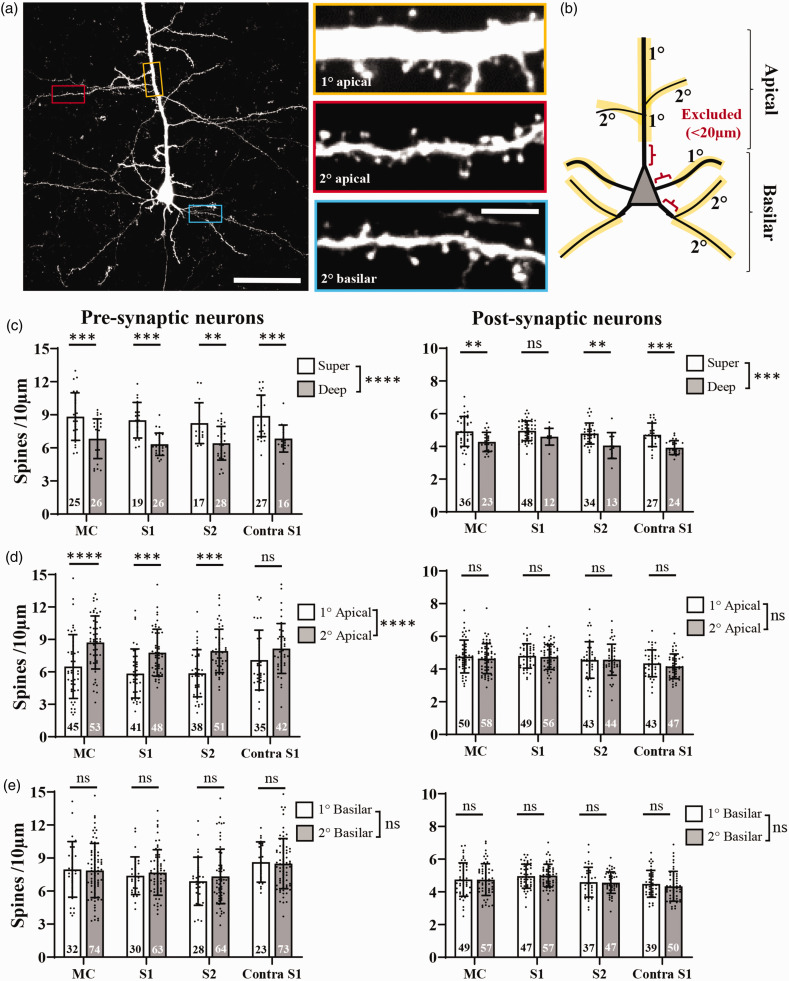
Dendritic spine density differences in control mice as a function of cortical depth or branch order in apical and basilar dendrites. (a) Confocal image showing GFP expression in retrogradely labelled layer 5 neuron in a sham control mouse. Insets show dendritic spines on different branches of the apical and basilar dendrite. (b) Schematic showing how dendritic branch orders were defined and zones of exclusion for our analysis. For graphs shown in c-e, pre-synaptic neurons are shown on the left (n = 11 sham mice), post-synaptic neurons on the right (n = 9 sham mice). (c) Graphs comparing spine density in superficial vs deep neurons across cortical regions (spine density in apical and basilar dendrites pooled). (d) Comparison of spine density in primary vs secondary apical dendrites in different cortical regions and (e) spine density in primary vs secondary basilar dendrites across cortical regions. The number of neurons sampled in each group is shown within each bar. Data in (c-e) analysed by 2-way ANOVA with Sidak’s multiple comparisons tests. Scale bars in a, left = 50 µm, insets = 5 µm. *p < 0.05, **p < 0.01, ***p < 0.001, ****p < 0.0001, ns: not-significant. Data presented as mean ± SD.

Before examining the effects of stroke on dendritic spines, we needed to determine if there were systematic differences in spine density based on whether neurons were located in superficial or deep cortical regions, and whether their dendrites were apical or basilar, primary or secondary. High-resolution image stacks were collected for 1 neuron at a time in our sham stroke controls (Figure 3(a)) and spine densities were quantified in primary and secondary apical and basilar dendrites, from superficial and deep cortical neurons, as shown in Figure 3(b). Since spines are extremely sparse next to the soma, we excluded the first 20 µm of dendrite from our analysis (Figure 3(b)). Spine densities in brains collected 1 or 6 weeks after sham stroke procedure were not significantly different and therefore pooled together to increase statistical power (Pre-synaptic neurons: F_(1,171)_=0.002, p = 0.96; Post-synaptic neurons: F_(1,209)_ = 1.59, p = 0.21). However, there were some systematic differences in dendritic spine density depending on neuronal depth from the surface or what branches were examined. For example, superficial neurons (defined as soma <450µm from pial surface) had significantly higher spine density than neurons in deeper cortical layers (Figure 3(c)), regardless of whether pre- or post-synaptic neurons were examined (Pre-synaptic neurons: F_(1,171)_ = 57.1, p < 0.0001; Post-synaptic neurons: F_(1,209)_ = 35.4, p < 0.0001). For apical dendrites, primary branches had significantly lower spine density than secondary branches in pre-synaptic neurons (Figure 3(d), left, F_(1,335)_ = 50.8, p < 0.0001), whereas there were no significant differences in post-synaptic neurons (Figure 3(d), right; F_(1,379)_ = 1.08, p = 0.30). Primary basilar dendritic spine densities were not significantly different from secondary basilar dendrites in both pre and post-synaptic neurons (Figure 3(e); Pre-synaptic neurons: F_(1,368)_ = 0.27, p = 0.60; Post-synaptic neurons: F_(1,366)_ = 0.13, p = 0.72). Given these systematic differences (or not), spine densities in subsequent figures will be shown separately for superficial and deep neurons, while primary and secondary basilar dendrites will be binned together. For apical dendrites, primary and secondary branches will be shown separately for pre-synaptic neurons or binned together for post-synaptic neurons.

### Effects of stroke on spine density in pre-synaptic neurons

**Figure 4.**
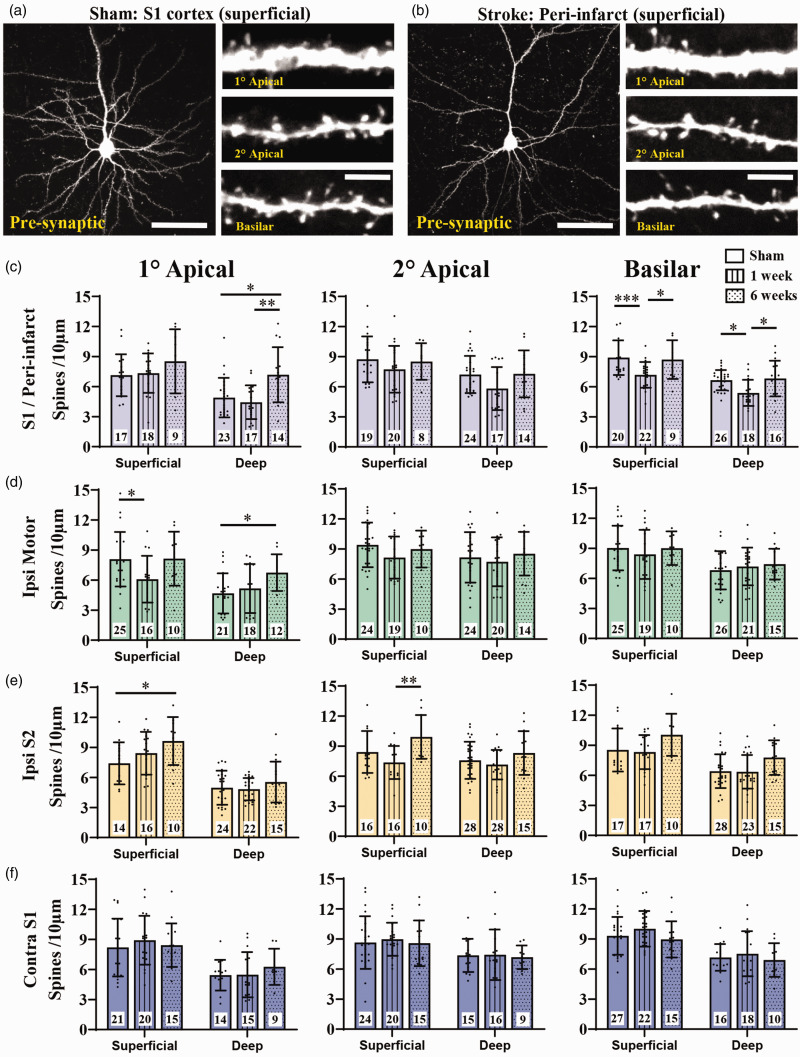
Effect of stroke on dendritic spine density in pre-synaptic neurons across cortical regions. (a) Confocal image showing a representative retrogradely labeled superficial neuron in S1 cortex in sham stroke mouse. (b) Confocal image of superficial neuron in peri-infarct cortex at 1 week recovery. Insets for a,b show higher magnification images apical and basilar branches. (c-f) Graphs show spine density for superficial and deep neurons in S1/peri-infarct cortex (c), ipsilateral motor cortex (d), ipsilateral S2 cortex (e) and contralateral S1 (f) as a function of apical and basilar branches. The number of neurons sampled within each group is shown within each bar. Neurons were obtained from the following number of mice: n = 11 shams, n = 8 one week stroke, n = 5 six week stroke. Data in (c-f) were analysed by 2-way ANOVA with Tukey’s multiple comparisons tests. Significant comparisons are denoted by asterisks, all other comparisons were not significant. *p < 0.05, **p < 0.01, ***p < 0.001. Scale bars in a,b left = 50 µm, insets = 5 µm. Data presented as mean ± SD.

Retrograde tracing of neurons that project to the FLS1 cortex (i.e. “pre-synaptic”) yielded bright fluorescently labeled neurons and dendritic spines (Figure 4(a) and (b)). For this analysis, we counted 100,642 spines from 444 neurons from 11, 8 and 5 mice from sham control, 1 and 6 weeks post-stroke, respectively. Based on these sample sizes and variances shown in Figure 4(c) to (f), effect sizes are shown in Supplementary Table 2 for each statistical comparison assuming an alpha level of 0.05 and beta of 0.2. One week after stroke, spine density in primary or secondary apical dendrites of peri-infarct neurons (<800µm of infarct border) did not change significantly (Figure 4(c), left, middle). However for peri-infarct basilar dendrites, spine density was significantly lower at 1 week for both superficial and deep neurons, which returned to normal values by 6 weeks recovery (Figure 4(c), right). Plotting apical or basilar spine density for each peri-infarct neuron as function of distance from the infarct border, revealed a trend for neurons closer to the infarct to have lower spine density (Supplementary Figure 3). Ipsilateral motor cortex neurons were sampled from a distance >800µm from the medial infarct border. At 1 week post-stroke, only superficial primary apical dendrites in motor cortex neurons exhibited a decrease in spine density (Figure 4(d)). Spine density in ipsilateral S2 and contralateral S1 neurons was not significantly altered at 1 week post-stroke (Figure 4(e) and (f)). Interestingly, spine density was significantly elevated above control levels at 6 weeks recovery in deep primary apical dendrites of peri-infarct and motor cortex neurons (Figure 4(c) and (d), left), as well as superficial S2 neurons (Figure 4(e), left). These findings suggest that dendritic spines on peri-infarct and motor cortex neurons that project to the stroke site are most likely to be affected by stroke.

Since it is conceivable that some retrogradely labelled cortical neurons in surviving brain regions may have undergone delayed cell death, a potential caveat of our single time point assessment of spine density at 7 days (post-stroke) is that our sample may be biased towards stroke resilient neurons. In order to estimate the extent of cell death in surviving and connected brain regions, we imaged retrogradely labelled neurons *in vivo* (208 neurons from 4 areas in 2 mice, mean distance from infarct border 0.63 ± 0.27 mm) before stroke and then again 2 and 4 days later. Tracking these neurons over time revealed that not a single neuron disappeared after stroke (Supplementary Figure 4). Consistent with previous research examining the effects of axotomy,^42^ these results suggest that stroke does not typically lead to cell death in cortical neurons in surviving brain regions (at least those ∼0.6 mm from the infarct border).

### Effects of stroke on spine density in post-synaptic neurons

**Figure 5.**
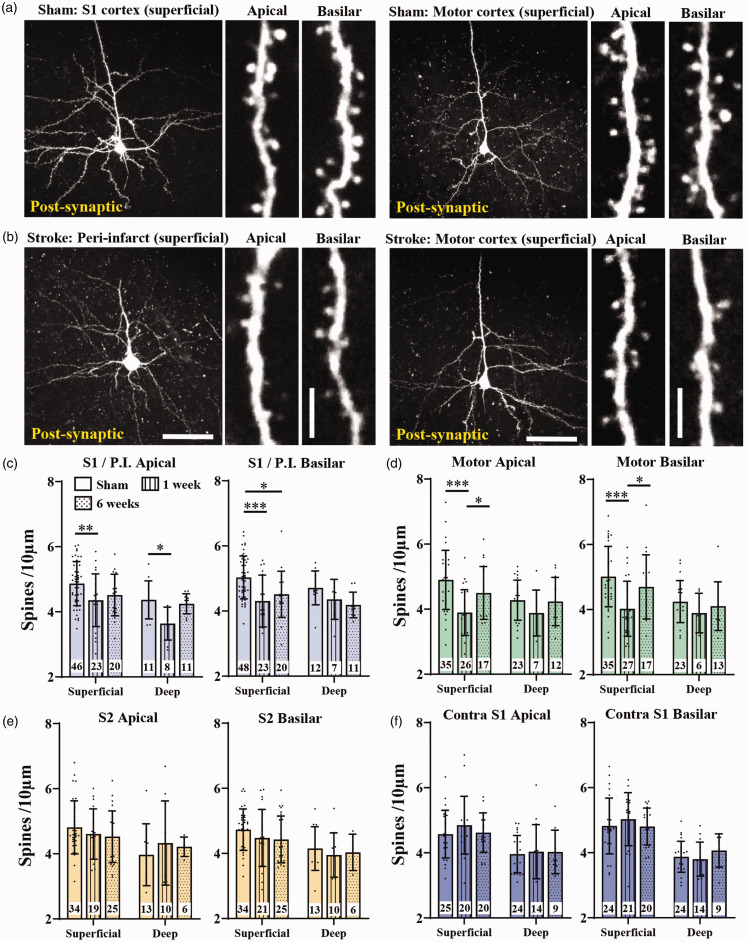
Effect of stroke on dendritic spine density in trans-synaptically labelled post-synaptic neurons in different cortical regions. (a) Two-photon images showing a representative post-synaptic superficial neuron in S1 and motor cortex in a sham stroke mouse. (b) Two-photon images of a superficial neuron in peri-infarct and motor cortex at 1 week recovery. Insets for a,b show higher magnification images apical and basilar branches. (c-f) Graphs show spine density for superficial and deep neurons in S1/peri-infarct cortex (c), ipsilateral motor cortex (d), ipsilateral S2 cortex (e) and contralateral S1 (f) as a function of apical and basilar branches. The number of neurons sampled within each group is shown within each bar. Neurons were obtained from the following number of mice: n = 9 shams, n = 10 one week stroke, n = 5 six week stroke. Data in (c-f) were analysed by 2-way ANOVA with Tukey’s multiple comparisons tests. Significant comparisons are denoted by asterisks, all other comparisons were not significant. *p < 0.05, **p < 0.01, ***p < 0.001. Scale bars in a,b left = 50 µm, insets = 5 µm. Data presented as mean ± SD.

Next we examined spine density in neurons post-synaptic to those in FLS1 cortex. Representative images of these trans-synaptically labelled neurons in S1/Peri-infarct and motor cortex are shown in Figure 5(a) and (b). For this experiment, we counted 73,389 spines in 470 neurons from 9, 10 and 5 mice from sham control, 1 and 6 weeks post-stroke, respectively. Based on these sample sizes and variances shown in Figure 5(c) to (f), we calculated effect sizes for each statistical comparison assuming an alpha level of 0.05 and beta of 0.2 (Supplementary Table 3). Spine density in peri-infarct neurons was significantly reduced 1 week after stroke for apical dendrites in both superficial and deep neurons (Figure 5(c)). Spine density was uniformly lower in peri-infarct neurons and did not change significantly as a function of distance from the infarct border (Supplementary Figure 5). Spine density in basilar peri-infarct dendrites was significantly reduced in superficial but not deep neurons (Figure 5(c)). Similarly, motor cortex neurons had significantly lower apical and basilar spine density in superficial neurons at 1 week recovery, whereas deep neurons were not affected (Figure 5(d)). In other connected regions such as ipsilateral S2 and contralateral S1, spine densities were unaffected by stroke (at both 1 and 6 weeks recovery) in both superficial and deep neurons (Figure 5(e) and (f)). Our data suggest that stroke has both local and more remote effects on post-synaptic neurons, particularly those in superficial peri-infarct and motor cortex.

## Discussion

Previous studies randomly sampling local and remote changes in dendritic spine density have yielded mixed and conflicting results. To help explain some of these differences, we examined dendritic spine density from multiple cortical regions that that either: a) project axons directly into the site of stroke damage (“pre-synaptic neurons”) or b) receive direct outputs from neurons destroyed by the stroke (“post-synaptic neurons”). For those neurons close to the stroke (peri-infarct region), we found a general reduction in dendritic spine density at 1 week recovery, which is consistent with several other studies that have examined spine density within the first few hours to days after stroke.^6,20,22,23^ However, our data extends previous observations which focused mainly on apical dendrites in peri-infarct layer 5 cortical neurons, in 2 important ways. First, we show that spine loss was particularly prominent in neurons that were post-synaptic to those destroyed by ischemic stroke, whereas stroke effects on pre-synaptic neurons were more nuanced and largely limited to basilar dendrites. Why this would occur remains speculative, although it is important to point out that post-synaptic peri-infarct neurons would not necessarily experience any direct ischemic damage to their axons, whereas axonal projections from pre-synaptic neurons would have necessarily been exposed to damaging stimuli in the infarct core. Since we show that none of the pre-synaptic neurons die off (Supplementary Figure 4), perhaps axonal damage could have triggered some resiliency or plasticity/repair programs that allowed them to buffer against the initial damage and/or promote recovery of spines after damage. This is not unheard of since brain slice preparations or transient ischemia can promote spine proliferation.^43–45^ Post-synaptic neurons on the other hand, may not have experienced direct damage but would have lost some of their inputs from FLS1 neurons destroyed by stroke. In this scenario, a loss of spines could reflect fewer FLS1 inputs (i.e. de-afferentation) since spines rarely exist without a pre-synaptic partner.

A second major extension of our study relative to previous ones, is that we examined spine density in neurons remotely connected to the infarct zone. Of note, superficial motor cortex neurons (both pre- and post-synaptic neurons) exhibited a significant loss of spines in the first week, which showed some recovery to normal levels by 6 weeks. The fact that superficial motor cortex neurons were more disrupted by stroke than deeper neurons could reflect subtle differences in the density or strength of cortico-cortical connections, whereby superficial motor neurons receive stronger inputs from somatosensory neurons than deep layer neurons.^46,47^ It is interesting to note that neurons in contralateral S1 were completely unaffected in the first week after stroke. While this finding may not hold for much larger ischemic strokes (e.g. damage to cortical and sub-cortical structures), our data are consistent with other papers examining focal cortical ischemia.^25,26^ Neurons in S2 cortex were also unaffected in the first week after stroke, which is somewhat surprising since FLS1 neurons normally send and receive dense connections with S2 cortex.^48,49^ However, primary apical dendrites from superficial S2 neurons that project to the FLS1 cortex (i.e. Pre-synaptic neurons, see Figure 4(e)), similar to that of deep peri-infarct and motor cortex neurons, showed a significant increase in spine density at 6 weeks post-stroke relative to sham controls. This structural finding in S2 neurons correlates with our previous voltage imaging studies showing uncharacteristically strong sensory evoked responses in superficial S2 cortex 3 months after focal stroke in FLS1 cortex.^39,50^ Perhaps the increased density of spines and functional activation in S2 neurons (and possibly other areas like motor cortex) reflects some progressive compensatory circuit activity or vicariation of function due to the loss of FLS1 neurons.^20,51,52^

There are limitations to our study that should be acknowledged. First, it is well known that AAVs have tropisms for certain cell types and could thus bias sampling. This property could explain why we had to restrict our analysis to select cortical regions since fluorescent labelling was unreliable in the thalamus, or certain contralateral regions. Moreover, our analysis focused on presumptive long-range excitatory projection neurons in layer 2/3 and 5 rather than interneurons or local circuits in layer 4. Second, although our study tried to equally sample male and female mice, it was underpowered to reliably detect sex differences in dendritic spine density after stroke. Since few studies have examined sex-dependent changes in spine density across cortical regions,^53^ despite well known sex differences in response to stroke,^54,55^ future studies could examine this issue more deeply. Third, while photothrombotic stroke can be targeted to specific locations which was essential for this study, we did encounter variability in the volume of infarction. This variability in ischemic damage could have influenced variability associated with spine density measurements, thus preventing us from detecting smaller differences between groups. However, we should point out that our effect sizes were generally large, ranging from 0.5–2.5 at 1 week recovery, which fits with previous effect size estimates.^56^ Further, our spine density changes (∼20% loss in specific areas) bear similarity to pre-clinical work showing 20–50% impairments in sensorimotor function after small cortical infarcts.^14,20,22,38,57–60^ Based on our observed spine density changes and effect sizes, we believe there is very likely a biologically meaningful difference between our experimental groups. What functional implications our findings represent is still unclear. The initial loss of spines in motor and peri-infarct regions, and its subsequent recovery over 6 weeks may partially explain forepaw deficits and recovery. Future studies could take advantage of AAVs that drive expression of optogenetic and chemogenetic actuators that allow silencing or stimulation of remotely connected neurons after stroke. This experiment could provide a more causal explanation for the long-standing question of what role the connectome (or its disruption, vis a vis diaschisis) plays in recovery of function after stroke.

## Supplemental Material

sj-pdf-1-jcb-10.1177_0271678X251345360 - Supplemental material for Retrograde and anterograde trans-synaptic viral tracing of neuronal connections reveals local and distant effects of ischemic stroke on dendritic spines Supplemental material, sj-pdf-1-jcb-10.1177_0271678X251345360 for Retrograde and anterograde trans-synaptic viral tracing of neuronal connections reveals local and distant effects of ischemic stroke on dendritic spines by Myrthe Van Sprengel, Jenna Butterworth, Patrick L Reeson and Craig E Brown in Journal of Cerebral Blood Flow & Metabolism
