# Supplementary material for: Retrograde and anterograde trans-synaptic viral tracing of neuronal connections reveals local and distant effects of ischemic stroke on dendritic spines
Source: J Cereb Blood Flow Metab. 2025 May 25;45(10):1891–904. doi: 10.1177/0271678X251345360 (PMC12106386; doi:10.1177/0271678X251345360)
Supplement: sj-pdf-1-jcb-10.1177_0271678X251345360 - Supplemental material for Retrograde and anterograde trans-synaptic viral tracing of neuronal connections reveals local and distant effects of ischemic stroke on dendritic spines [file sj-pdf-1-jcb-10.1177_0271678X251345360.pdf]

Supplementary Table 1: Summary of stroke affected animals used and source of attrition

ID	Retrograde vs Anterograde	Group	Reason for Exclusion
GK-N3	Retrograde	1 week	N/A
GN-N1	Retrograde	1 week	N/A
GK-N0	Retrograde	1 week	N/A
IB-N0	Retrograde	1 week	N/A
GR-N0	Retrograde	1 week	N/A
GP-N1	Retrograde	1 week	N/A
HC-N3	Retrograde	1 week	N/A
IC-N1	Retrograde	1 week	N/A
11-O6-3	Anterograde	1 week	N/A
11-O6-4	Anterograde	1 week	N/A
11-06-6	Anterograde	1 week	N/A
11-O8-3	Anterograde	1 week	N/A
I3-6	Anterograde	1 week	N/A
13-O4-4	Anterograde	1 week	N/A
13-O5b-1	Anterograde	1 week	N/A
13-O5b-4	Anterograde	1 week	N/A
G1-6	Anterograde	1 week	N/A
G1-9	Anterograde	1 week	N/A
GQ-N1	Retrograde	6 week	N/A
HA-N1	Retrograde	6 week	N/A
HA-N3	Retrograde	6 week	N/A
HW-N0	Retrograde	6 week	N/A
HW-N6	Retrograde	6 week	N/A
Km3	Anterograde	6 week	N/A
Km4	Anterograde	6 week	N/A
G4-3	Anterograde	6 week	N/A
G3-3	Anterograde	6 week	N/A
G3-6	Anterograde	6 week	N/A
GK-N1	Retrograde	1 week	Insufficient labelling
GK-N4	Retrograde	1 week	Insufficient labelling
GL-N0	Retrograde	1 week	Insufficient labelling
GM-N0	Retrograde	1 week	Insufficient labelling
GM-N1	Retrograde	1 week	Insufficient labelling
GM-N3	Retrograde	1 week	Insufficient labelling
GN-N0	Retrograde	1 week	Insufficient labelling
G0-N0	Retrograde	1 week	Insufficient labelling
G0-N3	Retrograde	1 week	Insufficient labelling
GT-N0	Retrograde	1 week	Insufficient labelling
IC-N0	Retrograde	1 week	Infarct not detected
GT-N1	Retrograde	6 week	Insufficient labelling
HD-N0	Retrograde	6 week	Insufficient labelling
HD-N1	Retrograde	6 week	Insufficient labelling
HW-N1	Retrograde	6 week	Improper stroke location
HW-N3	Retrograde	6 week	Improper stroke location

Supplementary Table 2. Effect sizes for <u>retrograde neurons</u> (alpha = 0.05 and Beta = 0.2)

		**Sham vs. 1 week**	**Sham vs. 6 weeks**	**1 vs 6 weeks**
**S1/peri-infarct**	1° Apical Superficial	1.923	2.701	2.598
	1° Apical Deep	1.635	2.082	2.093
	2° Apical Superficial	2.078	2.335	2.340
	2° Apical Deep	1.775	1.975	2.268
	Basilar Superficial	1.321	2.077	1.550
	Basilar Deep	0.991	1.241	1.482
**Ipsi Motor**	1° Apical Superficial	2.286	2.852	2.834
	1° Apical Deep	2.002	1.948	2.233
	2° Apical Superficial	1.859	2.137	2.153
	2° Apical Deep	2.099	2.209	2.252
	Basilar Superficial	2.042	2.047	2.263
	Basilar Deep	0.781	1.568	1.621
**Ipsi S2**	1° Apical Superficial	2.172	2.606	2.560
	1° Apical Deep	1.160	1.721	1.474
	2° Apical Superficial	1.849	2.412	2.160
	2° Apical Deep	1.310	1.812	1.698
	Basilar Superficial	1.848	2.371	2.120
	Basilar Deep	1.325	1.529	1.583
**Contra S1**	1° Apical Superficial	2.132	2.391	2.204
	1° Apical Deep	2.348	2.075	2.491
	2° Apical Superficial	1.816	2.260	1.880
	2° Apical Deep	2.105	1.675	2.160
	Basilar Superficial	1.475	1.670	1.682
	Basilar Deep	1.722	1.700	2.168

Supplementary Table 3. Effect sizes for <u>anterograde trans-synaptic</u> neurons (alpha = 0.05 and Beta = 0.2)

		**Sham vs. 1 week**	**Sham vs. 6 weeks**	**1 vs 6 weeks**
**S1/peri-infarct**	Apical Superficial	0.540	0.494	0.620
	Apical Deep	0.709	0.533	0.529
	Basilar Superficial	0.517	0.510	0.645
	Basilar Deep	0.755	0.534	0.683
**Ipsi Motor**	Apical Superficial	0.585	0.714	0.662
	Apical Deep	0.801	0.677	0.966
	Basilar Superficial	0.636	0.793	0.794
	Basilar Deep	0.804	0.678	0.937
**Ipsi S2**	Apical Superficial	0.637	0.593	0.668
	Apical Deep	1.321	0.863	1.150
	Basilar Superficial	0.588	0.500	0.661
	Basilar Deep	0.797	0.850	0.896
**Contra S1**	Apical Superficial	0.682	0.563	0.662
	Apical Deep	0.805	0.680	0.898
	Basilar Superficial	0.681	0.588	0.604
	Basilar Deep	0.471	0.541	0.618

Supplementary Figure 1

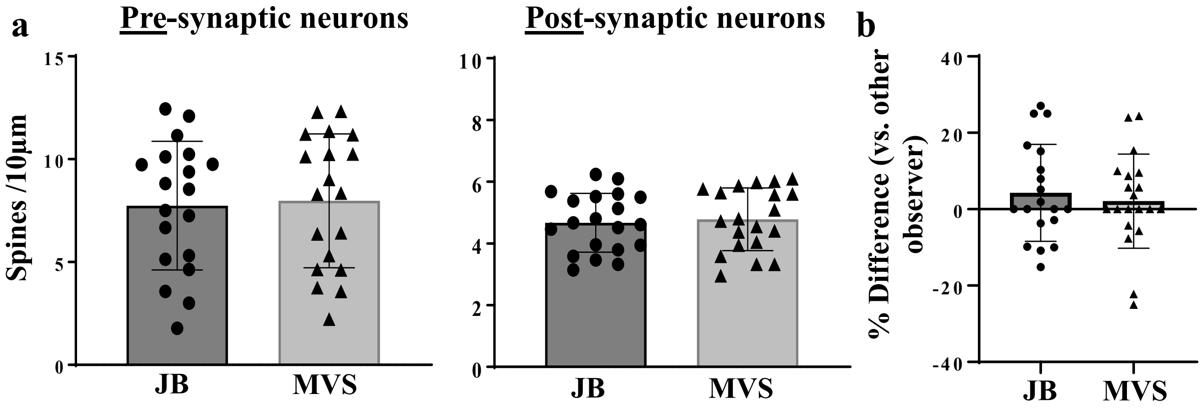

Supplementary Figure 1. (a) Comparison of spine density estimates from 20 neurons between 2 blinded observers (authors JB and MVS) for neurons pre-synaptic to FLS1 neurons or those post-synaptic. (b) Graph shows % difference in estimates between 2 observers. ns: not significant. Data in a,b were analysed with two-tailed unpaired t-tests. Data presented as mean ± SD.

Supplementary Figure 2

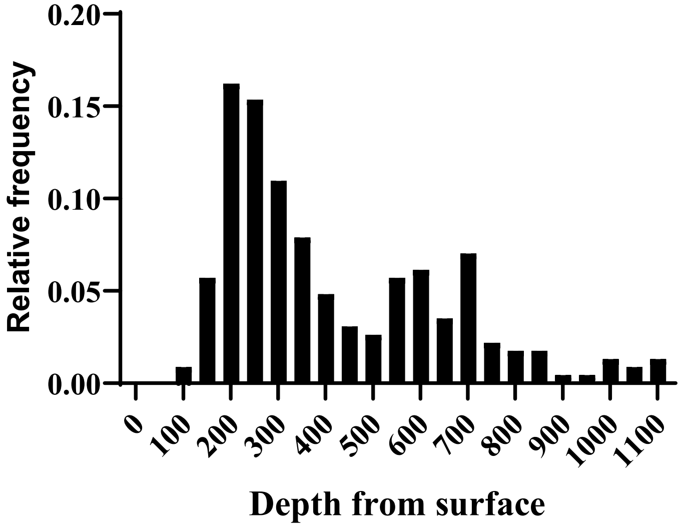

Supplementary Figure 2. Histogram showing the relative frequency of labelled neurons in sham controls as a function of cortical depth.

Supplementary Figure 3

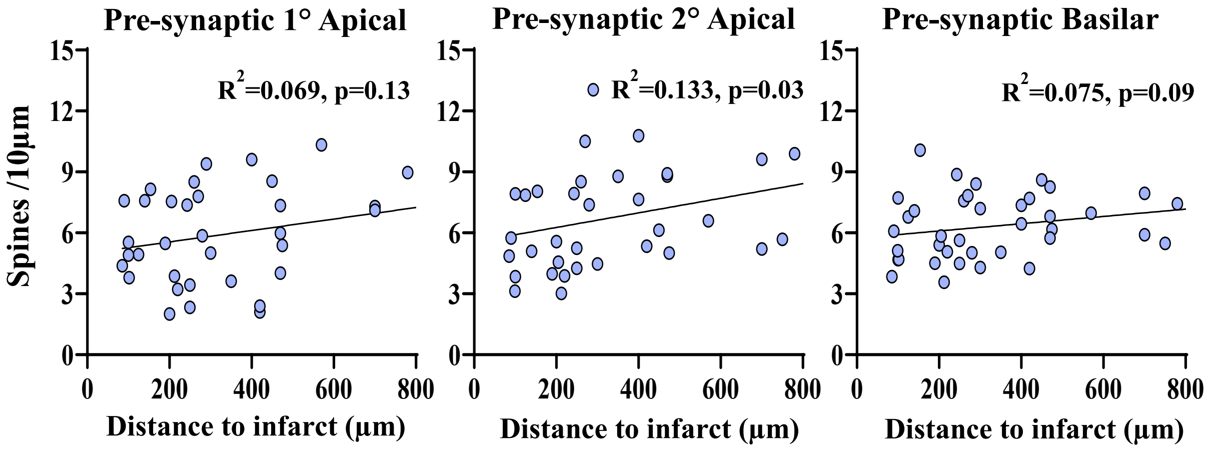

Supplementary Figure 3. Relationship between spine density in primary apical (left), secondary apical (middle) or basilar (right) dendrites in pre-synaptic peri-infarct neurons as a function distance from the infarct border. Data were analysed with linear regression.

Supplementary Figure 4

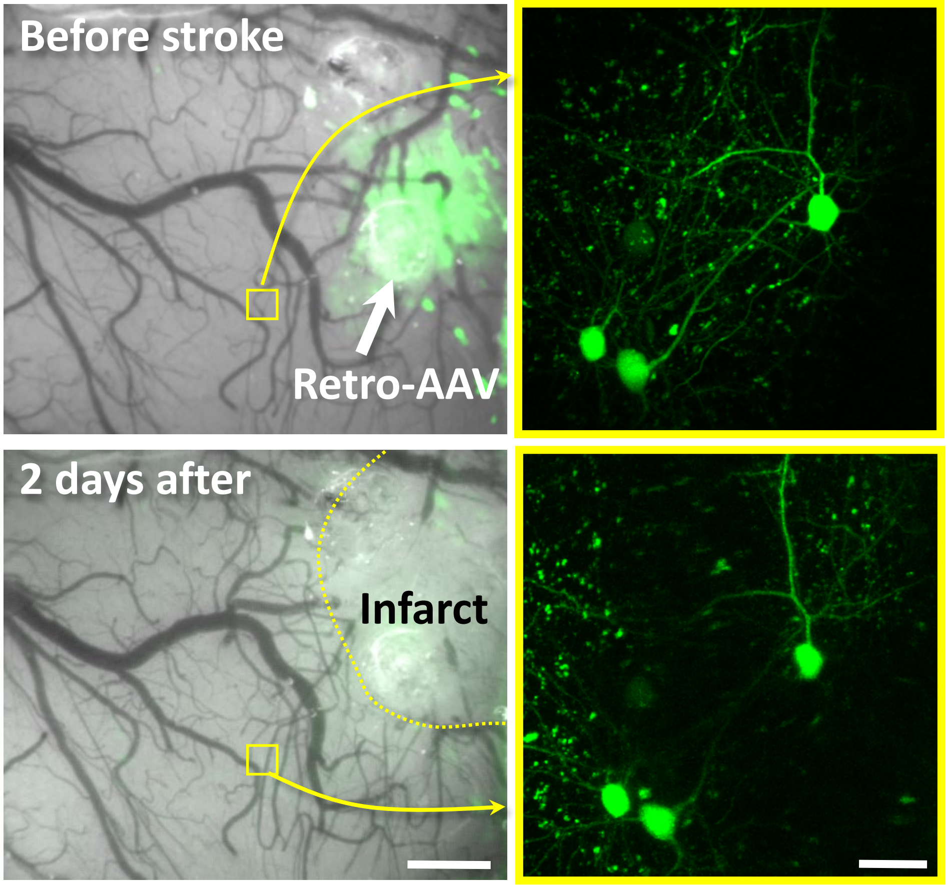

Supplementary Figure 4. Left column: Brightfield images of the cortical surface before and 2 days after focal stroke, were overlaid with images showing fluorescence associated with the injection of retro-AAV-GFP. Note the loss of GFP fluorescence in the infarct zone. **Right column:** Time lapse *in vivo* 2-photon images (max z-projection) showing the same neurons before and after stroke. Scale bars: 0.5mm (left) and 25µm (right).

Supplementary Figure 5

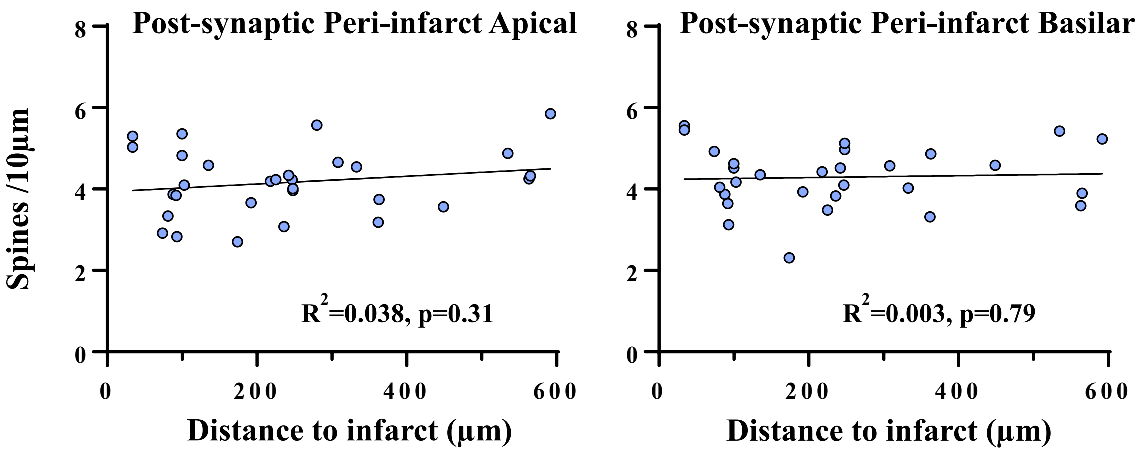

Supplementary Figure 5. Relationship between spine density in apical (left) or basilar (right) post-synaptic peri-infarct neurons as a function distance from the infarct border. Data were analysed with linear regression.
